# Rabies, host population structure, and cross-species transmission to the migratory bat *Tadarida brasiliensis* in Chile

**DOI:** 10.1371/journal.pntd.0013964

**Published:** 2026-02-19

**Authors:** Zulma E. Rojas-Sereno, Daniel G. Streicker, Verónica Yung, Alice Broos, Haris Malik, Laura Bergner, Michelle Lineros, Julio A. Benavides

**Affiliations:** 1 Instituto One Health y Doctorado en Medicina de la Conservación, Facultad Ciencias de la Vida, Universidad Andrés Bello, Santiago, Chile; 2 School of Biodiversity, One Health and Veterinary Medicine, University of Glasgow, Glasgow, United Kingdom; 3 MRC-University of Glasgow Centre for Virus Research, Glasgow, United Kingdom; 4 Sección Rabia, Departamento Laboratorio Biomédico, Instituto de Salud Pública de Chile, Santiago, Chile; 5 MIVEGEC, IRD, CNRS, Université de Montpellier, Montpellier, France; US Department of Agriculture, UNITED STATES OF AMERICA

## Abstract

Although many bat species migrate, the consequences of migration for bat population structure and rabies virus transmission remain poorly understood. Understanding the spatiotemporal dynamics of bat rabies (Lyssavirus rabies) transmission is important to prevent rabies spillovers to dead-end hosts, including humans. Here, focusing on the long-distance migratory Brazilian free-tailed bat (*Tadarida brasiliensis*) in South America, we identify the extent of host population and rabies virus structure across Chile. The analysis of 112 cytochrome b sequences from *T. brasiliensis* individuals and 144 rabies virus sequences submitted to the Chilean national rabies surveillance showed a lack of geographic clustering by municipality or region in Chile. This lack of clustering suggests that the migratory behavior of this species might be homogenizing the population structure of *T. brasiliensis* and its species-specific rabies virus (TbRV-SA). While most rabies virus sequences of *T. brasiliensis* (92%) corresponded to the TbRV-SA lineage, we also detected *T. brasiliensis* individuals infected by rabies viruses that are more strongly associated with other bats, including *Lasiurus spp.*, *Histiotus spp.,* and *Myotis spp* (8%)*.* In contrast, no other bat species were infected by TbRV-SA, suggesting the potential for a predominant asymmetric cross-species transmission observed in Chile. These results suggest that rabies risk could vary according to migratory bat movement patterns and community composition, especially in areas where cross-species transmission may be facilitated. Our study highlights the need to anticipate the spillover risk to humans and domestic animals associated with the long-distance spread of rabies virus in *T. brasiliensis*, and calls attention to clarify the role of urban areas as potential hotspots for cross-species transmission.

## Author summary

In this study, we explored how the long-distance movement of the Brazilian free-tailed bats can influence the spread of its rabies virus in Chile. This bat is known for traveling hundreds of kilometers, and we aimed to understand whether these movements influence the genetic distribution of this bat and of its rabies virus. We analyzed genetic data from both bats and the rabies virus they carried. Our results showed that the genetic structure of bats and their specific rabies virus were mixed across different Chilean regions, likely related to their migratory behavior. We also found that the Brazilian free-tailed bat was infected by the virus of the other three bat species, but it appeared not to transmit rabies to other insectivorous bats in our study area. Recognizing these patterns can help to understand and anticipate the spread of rabies virus and other pathogens in bats to humans and domestic animals.

## Introduction

Wildlife pathogens are influenced by host characteristics and population dynamics, including their migration (i.e., cyclic large-scale movements between habitat types), which can particularly influence the spatiotemporal distribution of a pathogen [1–3]. Migration can influence and facilitate the spread of bat viruses, including the introduction of new infected individuals to susceptible populations, or the alteration of contact rates between migratory and resident bats, facilitating cross-species transmission [2–6]. For example, the annual migration of the Straw-colored fruit bat (*Eidolon helvum)* has been linked to the occurrence of Ebola virus in humans in Africa [7]. Moreover, migration can affect host genetic structure and pathogen evolution [2,6,8]. Bat long-distance movements can enhance the levels of gene flow between populations of the same bat species, resulting in genetically similar populations. In contrast, population expansions can lead to the loss of rare alleles through genetic drift, creating spatial genetic structure [6,8–13]. Consequently, all these mechanisms resulting from different mixing of bat populations could affect the genetic structure of viruses carried by bats [2–6,14].

Understanding the correspondence between the genetic structure of bat populations and their viruses can provide insights into the spatiotemporal dynamics of rabies (Lyssavirus rabies) transmission in migratory bats [14–17]. Most spatio-temporal dynamics studies of rabies virus have focused on vampire bat dispersal in Latin America, given its predominant role affecting both livestock and humans, while the impact of bat migratory behavior on viral spread has received less attention [18–23]. Previous studies highlighted that female philopatry in the common vampire bat *Desmodus rotundus* creates a strong maternal (mtDNA) population structure, which was inconsistent with the relatively weak structure of vampire bat-associated rabies virus that is likely dispersed by males [17,24]. However, the correspondence between the genetic structure of bat populations and rabies virus has shown mixed results in other species with different ecological behaviors [13,14,17]. For example, rabies virus dispersal is influenced by the level of female philopatry in subpopulations of the migratory bat *Eptesicus fuscus* in North America [14,25]. In South America, migratory bat species such as *Tadarida brasiliensis* and *Lasiurus* spp. are considered the main rabies virus reservoirs to humans and companion animals [6,23,26–38]. However, despite the impact of these species on rabies virus transmission, the potential role of bat migration on rabies virus dynamics in South America remains unknown.

*T. brasiliensis* exhibits varying levels of migration across its range of distribution [6]. In northern bat populations, long-distance movements migrating from the USA to Mexico, and evidence of high fluctuation of females in the stopover roosts were documented [6,39–42]. Range expansions of *T. brasiliensis* have been observed in North America over the last two decades, likely driven by the expansion of suitable habitats for this species as a result of climate change [43,44]. In contrast, non-temperate neotropical populations are commonly considered non-migratory, although seasonal patterns of roost occupancy in Argentina suggest some level of migration [6,45,46]. Specific migratory routes are unknown in South America, but range expansions of *T. brasiliensis* have been observed, similar to northern populations [47,48]. Additionally, *T. brasiliensis* is highly adaptable to urban environments, forming large colonies in residential buildings with reports of a colony exceeding 60,000 individuals in the city of Rosario, Argentina [46,47,49–51]. Also, urban environments might facilitate interactions of *T. brasiliensis* with other bat species living in cities (e.g., *Lasiurus*, *Histiotus*, and *Myotis*), potentially enhancing cross-species transmission of pathogens [19,29,46,52]. But despite this spatial overlap, evidence of *T. brasiliensis* sharing roosts with other species in urban areas is limited to a few reports, including *Histiotus velatus* and *Molossus molossus* in Brazil, and *Myotis chiloensis* in Chile [19,46,52–55]. This combination of long-distance movements and ecological flexibility suggests a major involvement of *T. brasiliensis* in rabies virus circulation and cross-species transmission across South America.

*T. brasiliensis* is the main reservoir of rabies virus in humans and domestic animals in urban areas of Chile, with the circulation and transmission of *T. brasiliensis* rabies virus lineages in South America (TbRV-SA) [19,28]. The subcontinental movement of *T. brasiliensis* is likely to influence the genetic structure of its associated rabies virus variant (TbRV-SA), while urban habitats may lead to rabies cross-species transmission among Chilean bats. Given the variable migration patterns of *T. brasiliensis*, identifying its population structure could clarify the role of migration on the epidemiology and spillover risk of rabies. Therefore, this study aimed to (i) quantify the extent of the population structure in the widespread insectivorous bat *T. brasiliensis* in Chile using samples of dead *T. brasiliensis* submitted to the national rabies surveillance program, (ii) assess if the phylogeographic structure of the TbRV-SA matches *T. brasiliensis* population structure and (iii) evaluate the occurrence and spatial pattern of rabies virus transmission between insectivorous bats in Chile.

## Methods

### Ethics statement

Ethical approval for the sampling of dead bats was granted by the Ethics Committee of the Facultad Ciencias de la Vida, Universidad Andrés Bello, Santiago, Chile (036-2020).

### Bat sample collection

For our bat population genetic study, we collected 112 oral swab samples from dead *T. brasiliensis* bats that were submitted to the Chilean National Rabies Surveillance Program of the Instituto de Salud Pública (ISP, Santiago, Chile) from January to August 2022. The bats studied originated from 12 out of 16 Chilean administrative regions, from the northern Atacama to the southern Aysen regions, covering a range of 1887 km (S1 Fig). Each administrative region was assigned to one of the three geographical macrozones: northern, central, and southern. To account for a higher number of samples in the central zone, and following the classification system established by CORFO [56] and MinCiencia [57], we grouped samples by zones, including (i) the northern zone encompassing Big and Small North; (ii) the central zone comprising the Central Core, and the (iii) southern zone, including the South and Austral regions. Samples without geographic distribution were classified as non-reported (NR).

Municipalities were classified as rural or urban following the Chilean ‘Comisión Interministerial de Ciudad, Vivienda y Territorio-COMICIVY’T’ criteria (https://www.masvidarural.gob.cl/ruralidad-en-chile/). Municipalities were classified as urban if more than 75% of their population resides in areas with a population density exceeding 150 inhabitants per km^2^, and otherwise rural. Half (53%) of the bats included (59 out of 112) were obtained from the central zone and belonged mainly to urban municipalities (65%,73/112). Morphological identification of bats was carried out by experienced ISP staff. All bats sampled were rabies-negative according to the direct fluorescent antibody test. Swab samples were stored in RNAlater at -20°C in Chile until processed to characterize bat population structure using molecular methods. Inactivated DNA extractions from these oral swabs were sent to the University of Glasgow on dry ice to perform cytochrome b analyses.

### Amplification and sequencing of *T. brasiliensis* mitochondrial DNA

Nucleic acid extraction from oral swabs was performed with TRIzol reagent, using a modified version of the manufacturer’s protocol during the lysis and separation phases to optimize purification from swabs (Thermo Fischer Scientific, Waltham, MA, USA). The genetic structure of the bat population was analyzed using mitochondrial *cytochrome b*, following the protocol of Martins et al. [58]. PCR primers were designed to amplify 850 bp of cytochrome b (*CytB*) gene: TbCytB_F-5′-CGGCTCTCTCTTAGGAGTC-3′ and TbCytB_R-5′-CGGAAGGTTATGCTTCGTTG-3′. Sanger sequencing was performed by Eurofins Genomics, and samples were confirmed as *T. brasiliensis* using BLASTN in GenBank (http://blast.ncbi.nlm.nih.gov/, S1 File).

### Rabies virus sequence collection

Rabies virus sequence information from affected bats in Chile was collected using the RABV-Glue website (http://rabv-glue.cvr.gla.ac.uk/#/home). Our database contained one rabies virus whole genome sequence and two rabies virus glycoprotein sequences, while all other rabies virus sequences from bats in Chile (n = 144) represented the nucleoprotein (N) gene. Thus, we used sequences belonging to the N gene to study rabies virus population structure. The N gene is widely used in the analysis of geographic localization and species of origin for bat rabies virus, as reported in different bat studies [59–61]. A total of 98 rabies virus sequences from *T. brasiliensis* (*T. brasiliensis* Rabies Virus-South America (TbRV-SA) and 46 rabies virus sequences associated with other bat genera, including *Lasiurus* (*Lasiurus* Rabies Virus-South America (LRV-SA), 19 sequences), *Histiotus* (*Histiotus* Rabies Virus-South America (HtRV-SA), 18 sequences), and *Myotis* (*Myotis* Rabies Virus-South America (MyRV-SA), 9 sequences) were retrieved from GenBank. As GenBank includes only the year of publication and country of collection, additional information for each sequence was obtained from the ISP, including the municipality of origin and the year of sample collection. Rabies virus sequences represented 12 Chilean regions spanning 1392 km, extending from the Atacama to the Los Lagos regions. For *T. brasiliensis,* half of the rabies virus sequences (54%, 53/98) were obtained from the central zone, and the majority belonged to urban municipalities (85%, 83/98). In other bat species, most rabies virus sequences were also obtained from the central zone (65%, 30/46) and urban municipalities (72%, 33/46).

### Phylogenetic analyses

The *CytB* sequences from bats submitted to the ISP were aligned with sequences of *T. brasiliensis* from Chile (n = 14) and sequences from other countries, including the USA (n = 17), Bahamas (n = 34), and Brazil (n = 6) from GenBank (S1 Dataset). Sequences from Uruguay were excluded due to their shorter length. Consensus sequences were aligned using MUSCLE executed in MEGA 11.0.13. software (https://www.megasoftware.net/). The most likely evolutionary model was identified in IQ-TREE using ModelFinder Plus, based on the BIC score, and this model was subsequently applied for phylogenetic inference in IQ-TREE [62]. The substitution model identified for *CytB* sequences was HKY + F + G4. Bootstrapping analysis was run for 100000 replications. No branch collapsing was performed based on bootstrap support or branch length at any stage. Polytomies were identified using *is.binary* in ape package in R [64]. We confirmed that the *CytB* phylogenetic tree topology was fully bifurcating and that no nodes were collapsed during visualization. Consensus trees were visualized using FigTree v1.4.4 (http://tree.bio.ed.ac.uk/software/figtree/) and R 4.4.1 [63].

The rabies virus dataset was constructed with sequences obtained from the four bat-associated rabies virus lineages that circulate in Chile: TbRV-SA, LRV-SA, HtRV-SA, and MyRV-SA (S2 Dataset). Alignment and phylogenetic analyses for the rabies virus sequences followed the same methodology employed for *CytB* sequences. The substitution model identified for rabies virus sequences was TPM2 + F + G4. Polytomies were confirmed in the rabies virus phylogenetic tree topology.

### Clustering and statistical analyses

We visually assessed regional clustering by examining if sequences from specific regions or zones (north, central, south) clustered together within the reconstructed phylogenetic trees of *CytB* and rabies virus. To identify the effect of migration and population expansion on the genetic structure of *T. brasiliensis* population and whether the viral genetic structure was associated with geographic location, we estimated haplotype diversity, potential genetic structure, and tested the correlation between genetic and geographic distances for both *CytB* and rabies virus sequences in R-4.4.1. Haplotype diversity (H) and nucleotide diversity (N) were calculated using package pegas in R [65], with 10000 bootstrap replicates. Haplotype diversity was evaluated using the *haplotype* function, while nucleotide diversity was estimated using *nuc.div* function. The regional haplotype distribution was visualized by building a reconstructed haplotype network using the median-joining algorithm implemented in the *haploNet* function of the same R package.

To characterize the potential genetic structure associated with geographic location, we performed an analysis of similarities (ANOSIM) based on the results of a principal component analysis (PCA), utilizing the *dudi.pca* function from the adegenet package and the *anosim* function from the vegan package [66,67], with 10000 permutations. Finally, we constructed two pairwise distance matrices, one based on genetic distances among *CytB* sequences and the other based on geographic distances between sampling locations. Genetic matrix distances were estimated from aligned nucleotide sequences using the Kimura two-parameter model in the *dist.dna* function from the ape package [64]. Geographic matrix distances were estimated as great-circle distances from latitude and longitude coordinates using the *distm* function from the geosphere package [68]. To test the correlation between those genetic and geographic distance matrices, we conducted a Mantel test (10000 permutations) using the vegan package in R [67,69,70]. Sequence locations were estimated by assigning them the municipality centroid coordinates using QGIS 3.3.4 (QGIS, 2020). We also mapped and assessed the geographic distance between separating sequences from geographically distant regions that clustered together in the phylogenetic trees. Base layer map was obtained from the open-source site Global Administrative Areas (GADM) website (https://gadm.org/) using the geodata package in R [71].

To further explore the shared geographical genetic structure and characterize the relationship between *T. brasiliensis* population and its rabies lineage (TbRV-SA), we constructed bat host and virus phylogenies using the *cophylo* function from the phytools package in R [72]. Because host and virus sequences were not collected from the same individual bats (see methods on bat sampling and rabies sequences collection), a host-virus association data frame was compiled by randomly assigning a TbRV-SA sequence to a *T. brasiliensis* individual cytochrome b sequence from the same zone (n = 91 sequences).

Potential cross-species transmission was inferred if rabies virus sequences from a less predominant bat species were found within a lineage primarily composed of rabies virus sequences from a more predominant bat species (e.g., rabies virus sequences of *Lasiurus* spp. clustering together, with a single sequence from another bat species). To analyze the influence of urban versus rural landscapes on potential cross-species transmission within rabies virus lineages, we built a general linear mixed-effect model with a binomial residual distribution and a logit link function using the *glmer* function in R [73]. The model assessed the association between the presence/absence of potential cross-species transmission by a particular rabies virus lineage (e.g., bat infected by another bat lineage species, including TbRV-SA, LRV-SA, HtRV-SA, or MyRV-SA) and landscape type (urban or rural). Landscape was considered a fixed effect, while the administrative region was considered a random effect to account for potential spatial variation.

## Results

### Genetic structure of the *Tadarida brasiliensis* population

**Fig 1:**
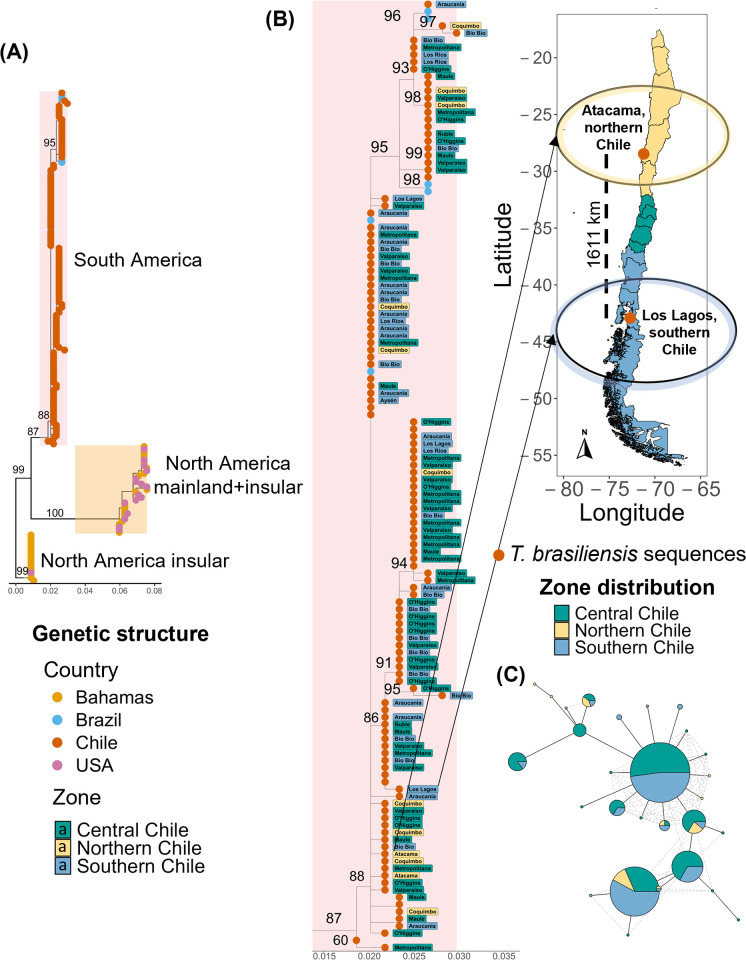
Genetic structure using cytochrome b (*CytB*) marker of *Tadarida brasiliensis* population. **(A).** The phylogenetic tree displays sequences for *T. brasiliensis CytB* in the Americas. Tip color represents the country of origin. **(B).** Cluster of *T. brasiliensis CytB* sequences in South America. Tip color labels indicate the three Chilean Zones, while text labels indicate the Chilean administrative regions. The map represents the location of *T. brasiliensis CytB* sequences that clustered together in the phylogenetic tree but had the farthest geographical distance between them. Base layer map was obtained from open-source site Global Administrative Areas (GADM) website (https://gadm.org/) using geodata package in R [71]. **(C).** Haplotype network for *T. brasiliensis CytB* sequences in Chile. Nodes represent unique haplotypes. The node size illustrates the haplotype frequency in the population, and colors refer to the percentage of that haplotype in each of the three Chilean zones. Edges indicate genetic distances between haplotypes, where shorter edges represent fewer mutations, while hash marks along edges represent inferred mutational steps between haplotypes.

The *CytB* phylogenetic tree for *T. brasiliensis* showed three independent clusters separating sequences from the Bahamas in North America (bootstrapping value (bv) = 99), mainland and insular North America (bv = 100), and South America (bv = 87, Fig 1A). In South America, sequences from Chile and Brazil were mixed in the same cluster, and no clustering by municipality or region was observed for Chilean sequences (Fig 1B). Sequences were distributed from the southern to northern zones of Chile, separated by a maximum approximate distance of 1611 km. Sequences from the northern zone (Atacama) were closely related to sequences from the southern zone (Araucania and Los Lagos).

We identified 28 haplotypes within the Chilean *T. brasiliensis* population, indicating high haplotype diversity (H = 0.9, 95% CI: 0.88–0.91, S2A and S2B Fig), but low nucleotide diversity (π = 0.005 ± 0.001). Haplotype diversity estimates by region were high (Central Chile: H = 0.9, 95% CI: 0.82–0.90, Southern Chile: H = 0.8, 95% CI: 0.70–0.88, Northern Chile: H = 0.8, 95% CI: 0.70–0.86), with the highest number of haplotypes in Central Chile (n = 18), and nucleotide diversity values remaining uniformly low (π = 0.004–0.005, S1 Table). However, four haplotypes accounted for almost 60% of the population, with a dominant central haplotype and few derived peripheral haplotypes forming a star-like structure in the haplotype network, suggesting recent population expansion (Fig 1C). Two of the most recent dominant haplotypes were shared across all regions. The PCA analysis revealed limited genetic structure, with the first principal component explaining only a small fraction (12%) of the genetic variation, while the plot showed a central point with radiating samples, but no distinct clusters (S2C Fig). Consistent with this observation, the ANOSIM test found no significant genetic differentiation among groups (R = 0.008, p-value = 0.172). Finally, pairwise genetic distances among *CytB* sequences were not correlated with pairwise geographic distances separating sampling locations (Mantel test, r = 0.0174, p = 0.340, S2D Fig).

### Genetic structure of rabies virus sequences from Chilean bats

**Fig 2:**
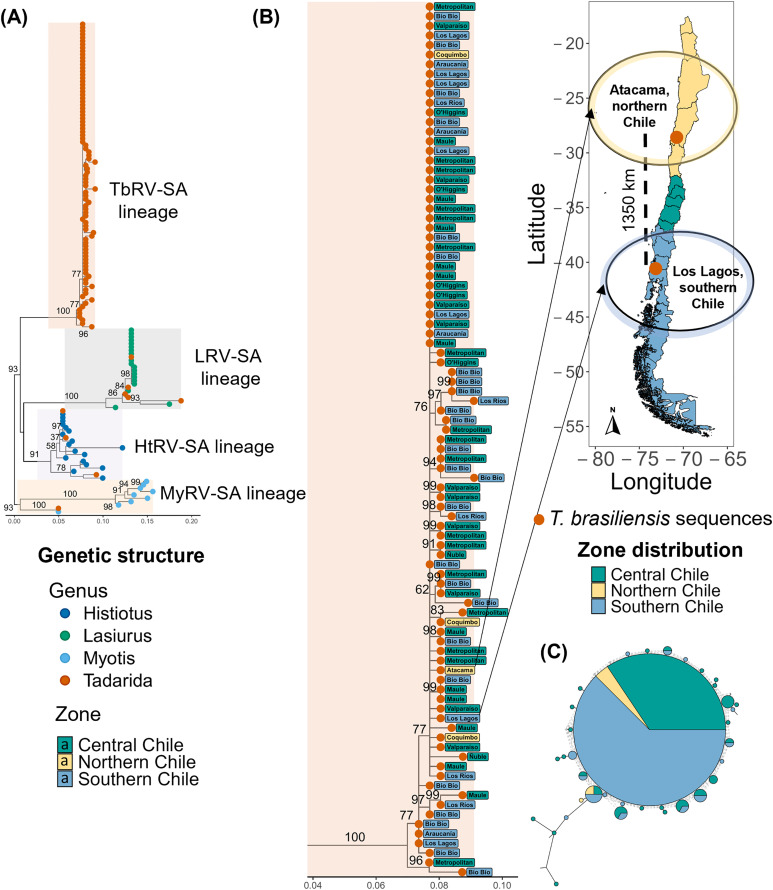
Genetic structure of the nucleoprotein *(N)* gene for rabies virus of *Tadarida brasiliensis* and other bat species in Chile. **(A).** The phylogenetic tree displays rabies virus sequences from *T. brasiliensis* and other bat species in Chile. Each color-shaded cluster represents a bat-rabies lineage: *T. brasiliensis* Rabies Virus-South America lineage (TbRV-SA, soft orange), *Lasiurus* Rabies Virus-South America lineage (LRV-SA, soft grey), *Histiotus* Rabies Virus-South America lineage (HtRV-SA, soft violet), *Myotis* Rabies Virus-South America lineage (MyRV-SA, soft yellow). The tip color represents the bat species sampled. **(B).** Lineage of *T. brasiliensis* Rabies Virus-South America (TbRV-SA) in Chile. Tip color labels indicate the three Chilean Zones, while text labels indicate the Chilean administrative regions. The map represents the location of TbRV-SA sequences that clustered together in the phylogenetic tree but had the farthest geographical distance between them. Base layer map was obtained from open-source site Global Administrative Areas (GADM) website (https://gadm.org/) using geodata package in R [71]. **(C).** Haplotype network for *T. brasiliensis* CytB sequences in Chile. Nodes represent unique haplotypes. The node size illustrates the haplotype frequency in the population, and colors refer to the percentage of that haplotype in each of the three Chilean zones. Edges indicate genetic distances between haplotypes, where shorter edges represent fewer mutations, while hash marks along edges represent inferred mutational steps between haplotypes.

As expected, the phylogenetic tree of rabies virus nucleoprotein sequences in Chile showed four distinct clusters, each associated with a specific bat genus (Fig 2A). We identified a viral lineage associated with *T. brasiliensis,* TbRV-SA (bv = 100), along with three other clusters (MyRV-SA, LRV-SA, HtRV-SA). Both TbRV-SA and MyRV-SA lineages were found in all zones, while LRV-SA and HtRV-SA were restricted to the Central and southern zones (S3A Fig). Interestingly, TbRV-SA sequences from the Atacama region in the north were closely related to sequences from the Los Lagos region in the south of Chile, spanning an approximate distance of 1350 km (Fig 2B).

Similar to the population structure of *T. brasiliensis*, TbRV-SA sequences included 45 haplotypes, indicating high haplotype diversity, where a dominant haplotype shared across all regions accounted for 38% of the viral population, and a few derived peripheral haplotypes (H = 0.9, 95% CI: 0.77–0.90, Figs 2C, S3B and S3C). Unlike other singletons that appeared isolated in the network, a small peripheral haplotype group was found in Southern Chile (blue, basal, first derived haplotype), with the presence of unique haplotypes that appear sequentially towards Central Chile (green, secondary derived haplotypes). Additionally, low nucleotide diversity (π = 0.02 ± 0.002) was also observed. Regionally, haplotype diversity values were moderate to high across Chilean zones (Central Chile: H = 0.8, 95% CI: 0.67–0.88, Southern Chile: H = 0.8, 95% CI: 0.66–0.88, Northern Chile: H = 0.7, 95% CI: 0.38–0.80), while low nucleotide diversity showed wide variation among regions (π = 0.005–0.02, S1 Table). In both analyses, the lowest diversity was found in Northern Chile (π = 0.02 ± 0.005). There was no clustering by municipality or administrative region within TbRV-SA phylogeny (ANOSIM test, R = 0.0008, p-value = 0.370, S3D Fig), and no significant correlation between the pairwise genetic and geographic distance matrices (r = -0.0458, p = 0.745, S3E Fig). The tanglegram showed a mixed pattern, without clusters by region (S4 Fig).

**Fig 3:**
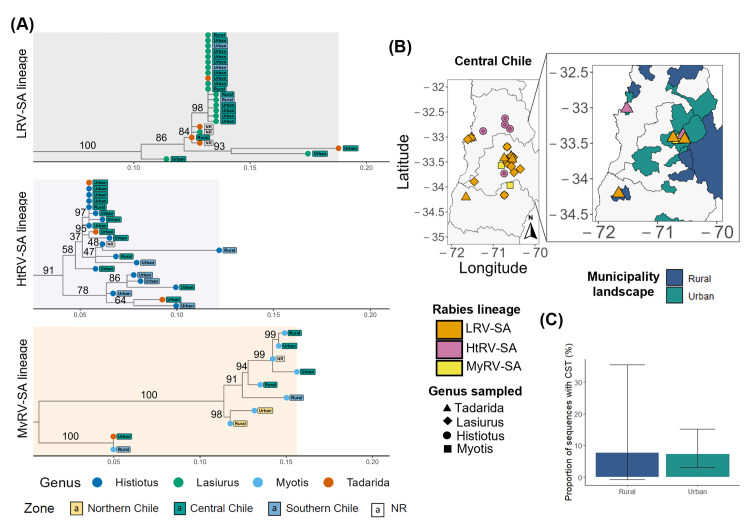
Genetic structure and spatial distribution of the *nucleoprotein (N)* gene sequences for non-*Tadarida* rabies virus in Chile. **(A).** Lineages of non-*Tadarida* rabies virus in Chile. Each figure section represents a bat-rabies lineage: *Lasiurus* Rabies Virus-South America lineage (LRV-SA, soft grey, top), cluster *Histiotus* Rabies Virus-South America lineage (HtRV-SA, soft violet, middle), cluster *Myotis* Rabies Virus-South America lineage (MyRV-SA, soft yellow, bottom). Tip color labels indicate the three Chilean Zones: northern (yellow), central (green), and southern (cyan-blue), while text labels indicate the municipality landscape. Samples without geographic distribution were classified as non-reported (NR). **(B).** Location of non-*Tadarida* rabies virus in Central Chile. The color of each geographic point represents the cluster belonging to a bat-rabies lineage: LRV-SA is strong orange, HtRV-SA is muted pink, and MyRV-SA is yellow. The shape represents the bat species sampled. The right map represents the location of non-*Tadarida* lineages of rabies virus in *T. brasiliensis* samples in central Chile according to their municipality landscape distribution. Municipality landscapes were categorized as rural (dark blue) or urban (dark green). Base layer map was obtained from open-source site Global Administrative Areas (GADM) website (https://gadm.org/) using geodata package in R [71]. **(C).** Proportion of sequences from *T. brasiliensis* bats involved in cross-species transmission (CST) according to the municipality landscape. Bars represent the proportion of sequences sampled from *T. brasiliensis* (Tb) per non-*Tadarida* lineage of rabies virus according to its municipality landscape distribution.

Rabies viruses associated with other bat species in Chile showed no evidence of geographic structure within their respective bat-rabies virus lineages. LRV-SA sequences (bv = 100) and MyRV-SA sequences (bv = 100) did not group by administrative region and formed separate clusters in the overall phylogenetic tree (Fig 3A). Rabies virus sequences from the HtRV-SA lineage (bv = 91) grouped into two clusters, with the top cluster including *Histiotus macrotus* rabies virus sequences found in both the central and the northern parts of the southern zone (bv = 58). The bottom cluster of HtRV-SA lineage (bv = 91) included *Histiotus* sp. sequences from the Magallanes region in the south and a unique sequence from the Metropolitan region in central Chile.

### Cross-species transmission of rabies viruses among Chilean bats

Each of the three phylogenetic lineages that were formed primarily of rabies virus sequences from non-*T. brasiliensis* bat species contained one or more sequences from *T. brasiliensis*, suggestive of cross-species transmission (Figs 3A, S5 and S2 Table). While most *T.* *brasiliensis* individuals (n = 91 out of 98, 92%) were infected by TbRV-SA, we also found individuals infected by rabies viruses more closely related to other bat species, including *Lasiurus* spp., *Histiotus* spp., and *Myotis* spp., and found predominately in central Chile (8%). Rabies viruses from other rabies virus bat lineages involved in apparent cross-species transmissions to *T. brasiliensis* were primarily detected in urban areas of the central zone; however, landscape type (urban or rural) was not statistically associated with the probability of cross-species transmission (rural: n = 1 out of 13 sequences infected by another bat lineage in rural areas, 7.7%, urban: n = 6 out of 83, 7.2%, Fisher p-value = > 0.05, GLMM, AIC = 61.8, Estimate = -18.83, p-value = 0.948, Fig 3B and 3C). However, within the MyRV-SA lineage, a rabies virus sequence of *T. brasiliensis* in the urban-central zone was closely related to *Myotis* sequences of the rural-southern zone and formed a separate cluster in the phylogeny (bv = 100, Fig 3A). In addition to the apparent transmission from other insectivorous bats to *T. brasiliensis*, the LRV-SA lineage contained sequences from both *Lasiurus borealis* and *L. cinereus* species, clustering together from the same and different administrative regions, suggesting transmission between these congeneric bat species. Notably, however, we detected no apparent transmission of TbRV-SA to other bat species.

## Discussion

Despite the established zoonotic risk posed by insectivorous bats, the relationship between the rabies virus and insectivorous bats remains poorly understood, especially outside of North America. Our analyses revealed a lack of significant geographic clustering in the genetic structure, characterized by a high haplotype diversity with dominant haplotypes shared across regions, but a low nucleotide diversity, for both *T. brasiliensis* and its associated rabies virus lineage (TbRV-SA) in Chile. These results suggest an extensive mobility of a recently expanded migratory population and potential widespread mixing of both bats and the rabies virus across Chile. Some *T. brasiliensis* rabies virus sequences (n = 6) from urban areas of central Chile clustered with sequences of rabies viruses from three other bat species (i.e., *Lasiurus spp.*, *Histiotus spp.,* and *Myotis spp.*). In contrast, no rabies virus sequences of other bat species were found within the cluster formed by sequences of TbRV-SA lineage. Despite our relatively limited sample size, these results suggest the potential for a predominant asymmetric cross-species transmission of rabies virus, where *T. brasiliensis* might acquire rabies virus from other bat species, with rare or no rabies virus transmission from *T. brasiliensis* to other bat species.

Our analysis of *T. brasiliensis* populations in Chile aligns with its expected migratory behavior and capacity for long-distance intracontinental flight, exceeding 1000 km. This is supported by previous records of *T. brasiliensis* traveling 1350 km and evidence of high levels of gene flow across populations in North America [6,8,9,13,74]. While *T. brasiliensis* is widely distributed in the Americas, the South American population is genetically differentiated from North American and Antillean groups [31,75]. Within Chile, low levels of population structure and high haplotype diversity, along with rare variants occurring and haplotypes shared across distant regions (more than 1000 km), suggest high connectivity, possibly facilitated by the absence of significant natural barriers. Although the Atacama Desert and the Andean mountains might constrain dispersal due to conditions unsuitable for *T. brasiliensis* [8,19,76–78], they do not appear to be decisive, as indicated by the levels of gene flow we observed extending into Brazil. Furthermore, rabies virus sequences from *Tadarida* in Chile and Argentina have been detected clustering together in previous studies [79], although we were not able to observe it in this study. Future research integrating genetic analyses and tracking ecology, such as radio frequency identification [80], could confirm the potential connectivity and exchange of individuals between both sides of the South American Andes.

Our analysis also suggests a recently expanded migratory population of *T. brasiliensis*, as evidenced by derived haplotypes stemming from four dominant haplotypes and limited nucleotide mutations accumulated, consistent with previous reports of colonization of new areas in Chile over the past decade [47]. Some intermediate haplotypes are missing in the network, likely representing unsampled or extinct variants resulting from drift or limited sampling. Although such expansions can reduce genetic diversity through genetic drift, high migration rates and expansion into ecologically similar habitats to the previous range likely minimize genetic differentiation by increasing gene exchange [8,11–13,81]. These processes may counteract potential bottlenecks in *T. brasiliensis* population by maintaining a high haplotypic diversity in the population, enhancing connectivity among populations, and promoting the spread of both common and rare variants across regions [8,9,11–13,81], as observed in our study.

Our phylogenetic analysis of the rabies virus nucleoprotein gene suggests that TbRV-SA in Chile is reflecting the long-distance movements, according to the population genetic structure of *T. brasiliensi*s. We therefore propose that *T. brasiliensis* migration across Chile likely reduces viral geographic genetic clustering by spreading the rabies virus across large distances. This hypothesis aligns with previous studies demonstrating how populations of insectivorous bats influence rabies virus genetic structure, such as *E. fuscus* connectivity influencing rabies virus structure in the USA and the role of *Miniopterus schreibersii* in Lyssavirus persistence in Spain [14,82]. An alternative explanation is a recent introduction of rabies virus into Chile; however, several observations argue against this hypothesis. Although rabies virus in both *D. rotundus* and *T. brasiliensis* has been reported in Latin America since the 1970s, *D. rotundus* rabies virus shows geographic clustering in Peru [17,27,28,83,84], suggesting sufficient time to cluster geographically in the absence of extensive geographic host movements. Additionally, high migration rates can counteract the effects of genetic drift and mutation in the virus [8], preventing regional genetic structure in rabies virus in Chile. However, the small peripheral haplotype group originated in Southern Chile, followed by the emergence of unique haplotypes from Central Chile, suggests a recent dispersion between southern and central zones. The small representation of these haplotypes could also be linked to the smaller sample size of sequences from southern and northern zones, which also reduces phylogenetic resolution and may generate unresolved nodes. Increasing sampling would therefore help to clarify whether a true geographic structure exists in TBRV-SA from *T. brasiliensis* located in Chilean populations.

We detected cross-species transmission of rabies virus from insectivorous bats (i.e., *Lasiurus* spp*.*, *Histiotus* spp*.,* and *Myotis* spp*.*) to *T. brasiliensis* in central Chile, but not in the opposite direction. Similar asymmetric and unidirectional cross-species transmission has been documented across the Americas, where geographic overlap is critical for spillover [50,79]. Although the small sample size in non-*Tadarida* species limits interpretation by reducing the statistical power to detect rare rabies transmission events from *T. brasiliensis*, our results still suggest a predominant asymmetry that may reflect host behavioral, viral, or ecological barriers. In central Chile, *Lasiurus* spp. and *T. brasiliensis* share foraging locations, leading to interspecific aggression (e.g., aerial chases) and subsequent viral exchange under limited resources [19,29,54,85–88]. *T. brasiliensis* also shares urban roosts with other bat species in the Americas, including the genus *Histiotus*, M*olossus*, and *Myotis* [52,55]. One possible explanation for this asymmetric transmission despite overlap is that solitary or small-colony bat species require higher aggression to maintain rabies virus circulation than large colonies of *T. brasiliensis*. Although niche preferences might limit aggression in non-rabid bats, as described in mixed urban colonies with *M. chiloensis* in Chile [54,89], the lack of behavioral studies on rabid bats (e.g., whether infection alters aggression towards other species) limits our ability to support this hypothesis. An alternative explanation is that bat community composition shapes patterns of CST, such as communities with a greater presence of closely related species that facilitate the transmission [50,55]. This interpretation is supported by reports of *T. brasiliensis* transmitting rabies to other molossid bat species in South America [79], suggesting that CST within this family may occur more frequently than previously recognized for TBRV-SA. Among Chilean molossids, only *T. brasiliensis* is widely distributed, and the preference for urban habitat may reduce the overlap with other species of the same family, which are more commonly distributed in natural areas of the northern regions in Chile [51,90], suggesting a limited geographical overlap to promote cross-species rabies transmission.

To our knowledge, this is the first study to explore the genetic structure of both bat hosts and rabies virus in Chile. Nonetheless, several limitations should be addressed by future research. First, although large-scale bat seasonal migration may influence bat rabies virus structure in Chile, local dispersal (e.g., sex-biased dispersal) also affects the local spread within and between species [14,17]. Our ability to assess migration, expansion, and dispersal was limited by the use of mitochondrial markers, which have slower evolutionary rates than other nuclear markers, and only infer maternal inheritance [14,17,25]. Therefore, our results could be considered as partial evidence that migration contributes to the mixed genetic structure observed in *T. brasiliensis* across Chile, which should be further tested using nuclear markers. Moreover, while the patterns of genetic structure observed in this study on cytochrome b and rabies virus in *T. brasiliensis* are consistent with previous findings on the role of females in North American insectivorous bats [14], the potential role of males in rabies virus dynamics should not be overlooked. Similarly, for rabies virus, the star-like haplotype network and lack of geographic clustering could reflect true expansion events driven by host migration but might also be an artifact of limited resolution in the nucleoprotein gene. Although the nucleoprotein gene of rabies virus has been successfully used to identify the geographical clustering of rabies virus among bats [17,29,84], other molecular methods could enhance fine-scale genetic structure. Therefore, future studies should incorporate rapidly evolving markers (e.g., microsatellites and single-nucleotide polymorphisms), which can offer more resolution to detect recent events of expansion, and could also elucidate potential sex-biased dispersal or migration alongside mitochondrial data [14,15,17,25,75,91,92]. Additionally, whole-genome sequencing could provide higher resolution to distinguish whether rabies lineage patterns result from multiple viral introductions, recent expansions, or marker constraints.

A second limitation is the use of samples derived from dead bats collected through passive surveillance, which influences the spatial and temporal distribution of bats and rabies sequences, potentially introducing bias in the spatio-temporal patterns obtained. For example, lower reports of a given Chilean region or municipality areas, as well as lower reports of certain bat species rarely interacting with humans, could limit inference regarding the geographic diversity of bats and rabies, as well as the detection of cross-species transmission between *Tadarida* and non-*Tadarida* species. The restricted sample size, particularly in the southern and northern regions of Chile, for both bats and viral sequences, likely reflects the lower number of dead bats reported from regions and municipalities that are far from the main ISP office located in Central Chile (Santiago capital). Finally, the lack of molecular identification of non-*T. brasiliensis* bats, which is not implemented in the ISP surveillance system, could also limit the inference of cross-species transmission. While the relatively low bat diversity in Chile (14 species) minimizes potential misclassification errors, this applied particularly at the genus level [51,93]. For example, uncertainty in bat identification below the genus level precluded the evaluation of clustering within the genus *Histiotus*, making it difficult to probe whether separate sections in the *Histiotus* lineage correspond to different bat sampled species or reflect regional isolation in a non-migratory genus. This also implies that any apparent lack of genetic structure in the non-*Tadarida* species should be interpreted with caution, given the limited sample sizes, the absence of molecular species identification, and their differences in geographic ranges. While migratory genera might display similar patterns to *Tadarida* (e.g., *Lasiurus*), non-migratory species such as *M. chiloensis* could show regional clustering [6,77], which was not detected in our results. As a complement to routine passive surveillance, active sampling of different bat species across regions, roost ecology studies (e.g., co-rooting, roost site fidelity), and molecular identification of bat species could improve our understanding of rabies in underrepresented regions.

Our findings suggest connectivity among *T. brasiliensis* populations across Chile, which could also facilitate the geographic spread of other viruses infecting *T. brasiliensis*, such as herpesvirus and adenovirus, both known to circulate in bats [15,16]. Alphacoronaviruses have also been reported to be shared between *T. brasiliensis, Myotis,* and molossid bats in mixed colonies, raising the possibility that cross-species pathogen transmission to *T. brasiliensis* could subsequently spread across Chile following *T. brasiliensis* movements [55,94,95]. If *T. brasiliensis* populations are indeed connected across South America, they could spread the rabies virus and other pathogens at the continental level, as suggested by the low RABV genetic distances among the TbRV-SA, when compared to other species-specific bat-RABV lineages [79]. Additional sequencing of bat rabies viruses from across Latin America would be valuable to understand how host phylogeny, ecological overlap, and possibly anthropogenic change influence pathogen spread and CST patterns.

## Supporting information

S1 Fig Distribution of *Tadarida brasiliensis’* oral swab samples and rabies virus sequences in Chile. (**A**). Oral swab samples of *T. brasiliensis* submitted to the Chilean National Rabies Surveillance Program of the Instituto de Salud Pública (ISP) distributed in the sampled municipalities (dark green), while bars represent the total number of samples by administrative region in Chile. Base layer map was obtained from the open-source site Global Administrative Areas (GADM) website (https://gadm.org/) using geodata package in R [71]. (**B**). Chilean rabies virus sequences were obtained from GenBank distributed in the municipalities of origin, while bars represent the total number of sequences by administrative region in Chile. Chilean zones were divided into northern (yellow), central (green), and southern (cyan-blue).
(PDF)

S2 Fig Genetic analyses of *Tadarida brasiliensis* cytochrome b (*CytB*). (**A**) Bars represent haplotype frequency. (**B**). Bars represent the bootstrap distribution of haplotypic diversity. Dashed lines in blue represent 95% confidence intervals. Dashed red line represents the mean bootstrap value (bv = 0.89, IC 95% = 0.86-0.91). (**C**). Discriminant analysis of principal components for *Tadarida brasiliensis* cytochrome b (*CytB*). The main plot demonstrates the first two discriminant axes. Color represents Chilean Zones. The secondary plot inserted in the top left displays the percentage of variation by component. (**D**). Correlation between pairwise genetic distance and pairwise geographic distance for *T. brasiliensis* cytochrome b (*CytB*). The red line represents the tendency of the correlation.
(PDF)

S3 Fig Regional distribution and genetic analyses of *Tadarida brasiliensis* of rabies lineages in Chile. (**A**) Bars represent the total number of rabies virus sequences per bat-rabies lineage by zone. Each bar represents a bat-rabies lineage: *Tadarida* Rabies Virus-South America (TbRV-SA), *Lasiurus* Rabies Virus-South America (LRV-SA), *Histiotus* Rabies Virus-South America (HtRV-SA), *Myotis* Rabies Virus-South America (MyRV-SA). Chilean zones were divided into northern (yellow), central (green), and southern (cyan-blue). Samples without geographic distribution were classified as non-reported (NR, grey). (**B**). Bars represent haplotype frequency. (**C**). Bars represent the bootstrap distribution of haplotypic diversity. Dashed lines in blue represent the 95% confidence intervals. The dashed red line represents the mean bootstrap value (bv = 0.84, IC 95% = 0.77-0.90). (**D**). Discriminant analysis of principal components for *T. brasiliensis* rabies virus sequences. The main plot demonstrates the first two discriminant axes (ANOSIM test, R = 0.0008, p-value = 0.370). Color represents Chilean Zones. The secondary plot inserted in the top left displays the percentage of variation by component. (**E**). Correlation between pairwise genetic distances and pairwise geographic distances for *T. brasiliensis* rabies virus. The red line represents the tendency of the correlation.
(PDF)

S4 Fig Tanglegram shows the relationship between *T. brasiliensis* cytochrome b (TbCytB) and *Tadarida* Rabies Virus-South America (TbRV-SA) sequences. Links connect each TbRV-SA sequence to a *T. brasiliensis* cytochrome b sequence from the same Chilean zone. Link colors indicate the Chilean zone of origin. Chilean zones were divided into northern (yellow), central (green), and southern (cyan-blue).
(PDF)

S5 Fig Location of non-*Tadarida* rabies virus in Chile. **(A)**. *Lasiurus* Rabies Virus-South America (LRV-SA) lineage sample distribution. **(B)**. *Myotis* Rabies Virus-South America (MyRV-SA) lineage sample distribution. **(C)**. *Histiotus* Rabies Virus-South America (HtRV-SA) lineage sample distribution. The dot color and shape represent the bat species sampled and zone distribution. Chilean zones were divided into northern (yellow), central (green), and southern (cyan-blue). Base layer map was obtained from the open-source site Global Administrative Areas (GADM) website (https://gadm.org/) using geodata package in R [71].
(PDF)

S1 Table Distribution of *Tadarida* and non-*Tadarida* rabies virus in Chile. H: haplotype diversity, CI: Confidence interval, N: nucleotide diversity, SD: standard deviation, TbRV-SA: *Tadarida* Rabies Virus-South America.
(PDF)

S2 Table Distribution of *Tadarida* and non-*Tadarida* rabies virus in Chile. HtRV-SA: *Histiotus* Rabies Virus-South America, LRV-SA: *Lasiurus* Rabies Virus-South America, MyRV-SA: *Myotis* Rabies Virus-South America, TbRV-SA: *T. brasiliensis* Rabies Virus-South America. Samples without geographic distribution were classified as non-reported (NR).
(PDF)

S1 Dataset Supporting data for *Tadarida brasiliensis* cytochrome-b sequences. (CSV)

S2 Dataset Supporting data for rabies virus sequences from Chile. (CSV)

S1 File Cytochrome-b sequences of *Tadarida brasiliensis* from Chile. (FASTA)
