# Supplementary material for: Rabies, host population structure, and cross-species transmission to the migratory bat Tadarida brasiliensis in Chile
Source: PLoS Negl Trop Dis. 2026 Feb 19;20(2):e0013964. doi: 10.1371/journal.pntd.0013964 (PMC12919816; doi:10.1371/journal.pntd.0013964)
Supplement: S1 Fig — (A). Oral swab samples of T. brasiliensis submitted to the Chilean National Rabies Surveillance Program of the Instituto de Salud Pública (ISP) distributed in the sampled municipalities (dark green), while bars represent the total number of samples by administrative region in Chile. Base layer map was obtained from the open-source site Global Administrative Areas (GADM) website (https://gadm.org/) using geodata package in R [71]. (B). Chilean rabies virus sequences were obtained from GenBank distributed in the municipalities of origin, while bars represent the total number of sequences by administrative region in Chile. Chilean zones were divided into northern (yellow), central (green), and southern (cyan-blue). (PDF) [file pntd.0013964.s001.pdf]

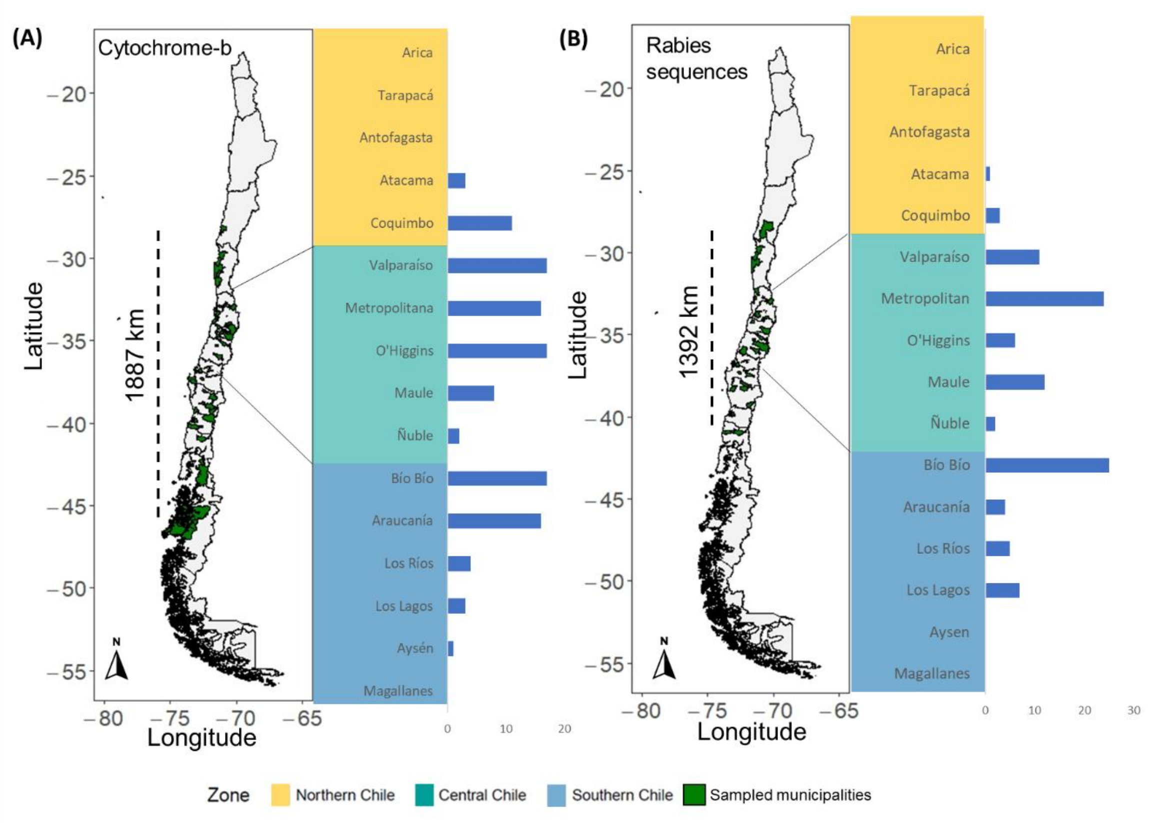

S1 Fig. Distribution of *Tadarida brasiliensis*' oral swab samples and rabies virus sequences in Chile. (**A**). Oral swab samples of *T. brasiliensis* submitted to the Chilean National Rabies Surveillance Program of the Instituto de Salud Pública (ISP) distributed in the sampled municipalities (dark green), while bars represent the total number of samples by administrative region in Chile. Base layer map was obtained from the open-source site Global Administrative Areas (GADM) website (https://gadm.org/) using geodata package in R [71]. (**B**). Chilean rabies virus sequences were obtained from GenBank distributed in the municipalities of origin, while bars represent the total number of sequences by administrative region in Chile. Chilean zones were divided into northern (yellow), central (green), and southern (cyan-blue).
