# Supplementary material for: Rabies, host population structure, and cross-species transmission to the migratory bat Tadarida brasiliensis in Chile
Source: PLoS Negl Trop Dis. 2026 Feb 19;20(2):e0013964. doi: 10.1371/journal.pntd.0013964 (PMC12919816; doi:10.1371/journal.pntd.0013964)
Supplement: S2 Fig — (A) Bars represent haplotype frequency. (B). Bars represent the bootstrap distribution of haplotypic diversity. Dashed lines in blue represent 95% confidence intervals. Dashed red line represents the mean bootstrap value (bv = 0.89, IC 95% = 0.86-0.91). (C). Discriminant analysis of principal components for Tadarida brasiliensis cytochrome b (CytB). The main plot demonstrates the first two discriminant axes. Color represents Chilean Zones. The secondary plot inserted in the top left displays the percentage of variation by component. (D). Correlation between pairwise genetic distance and pairwise geographic distance for T. brasiliensis cytochrome b (CytB). The red line represents the tendency of the correlation. (PDF) [file pntd.0013964.s002.pdf]

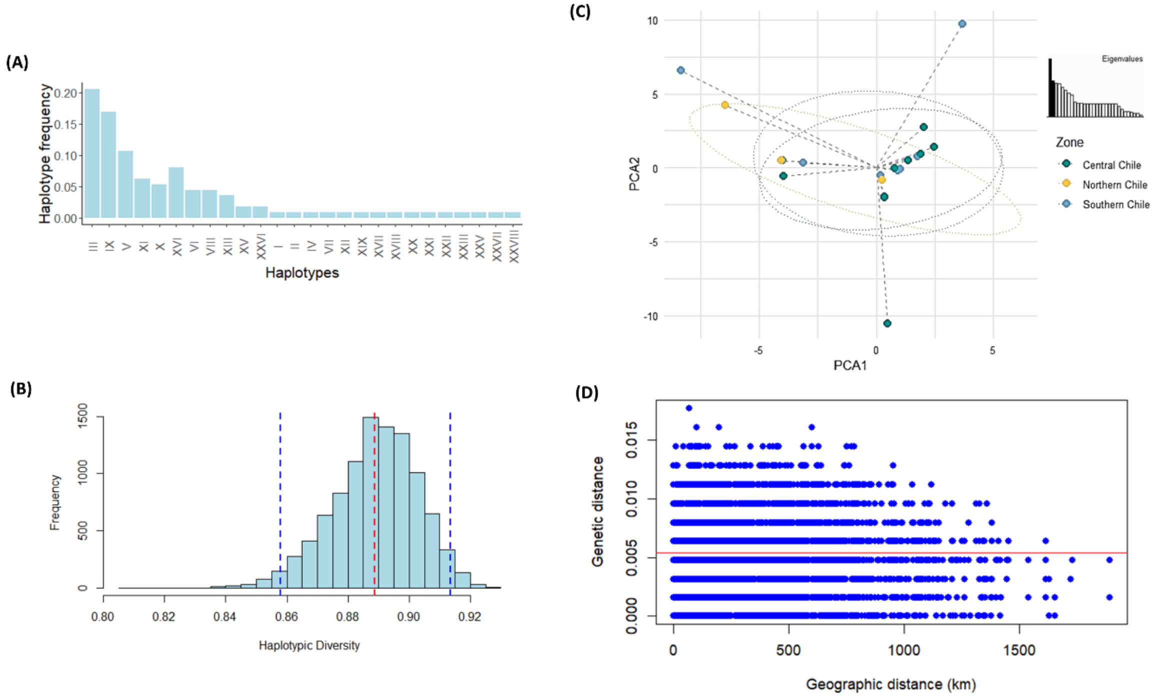

S2 Fig. Genetic analyses of *Tadarida brasiliensis* cytochrome b (*CytB*). (**A**) Bars represent haplotype frequency. (**B**). Bars represent the bootstrap distribution of haplotypic diversity. Dashed lines in blue represent 95% confidence intervals. Dashed red line represents the mean bootstrap value (bv = 0.89, IC 95%= 0.86-0.91). (**C**). Discriminant analysis of principal components for *Tadarida brasiliensis* cytochrome b (*CytB*). The main plot demonstrates the first two discriminant axes. Color represents Chilean Zones. The secondary plot inserted in the top left displays the percentage of variation by component. (**D**). Correlation between pairwise genetic distance and pairwise geographic distance for *T. brasiliensis* cytochrome b (*CytB*). The red line represents the tendency of the correlation.
