# Supplementary material for: Rabies, host population structure, and cross-species transmission to the migratory bat Tadarida brasiliensis in Chile
Source: PLoS Negl Trop Dis. 2026 Feb 19;20(2):e0013964. doi: 10.1371/journal.pntd.0013964 (PMC12919816; doi:10.1371/journal.pntd.0013964)
Supplement: S3 Fig — (A) Bars represent the total number of rabies virus sequences per bat-rabies lineage by zone. Each bar represents a bat-rabies lineage: Tadarida Rabies Virus-South America (TbRV-SA), Lasiurus Rabies Virus-South America (LRV-SA), Histiotus Rabies Virus-South America (HtRV-SA), Myotis Rabies Virus-South America (MyRV-SA). Chilean zones were divided into northern (yellow), central (green), and southern (cyan-blue). Samples without geographic distribution were classified as non-reported (NR, grey). (B). Bars represent haplotype frequency. (C). Bars represent the bootstrap distribution of haplotypic diversity. Dashed lines in blue represent the 95% confidence intervals. The dashed red line represents the mean bootstrap value (bv = 0.84, IC 95% = 0.77-0.90). (D). Discriminant analysis of principal components for T. brasiliensis rabies virus sequences. The main plot demonstrates the first two discriminant axes (ANOSIM test, R = 0.0008, p-value = 0.370). Color represents Chilean Zones. The secondary plot inserted in the top left displays the percentage of variation by component. (E). Correlation between pairwise genetic distances and pairwise geographic distances for T. brasiliensis rabies virus. The red line represents the tendency of the correlation. (PDF) [file pntd.0013964.s003.pdf]

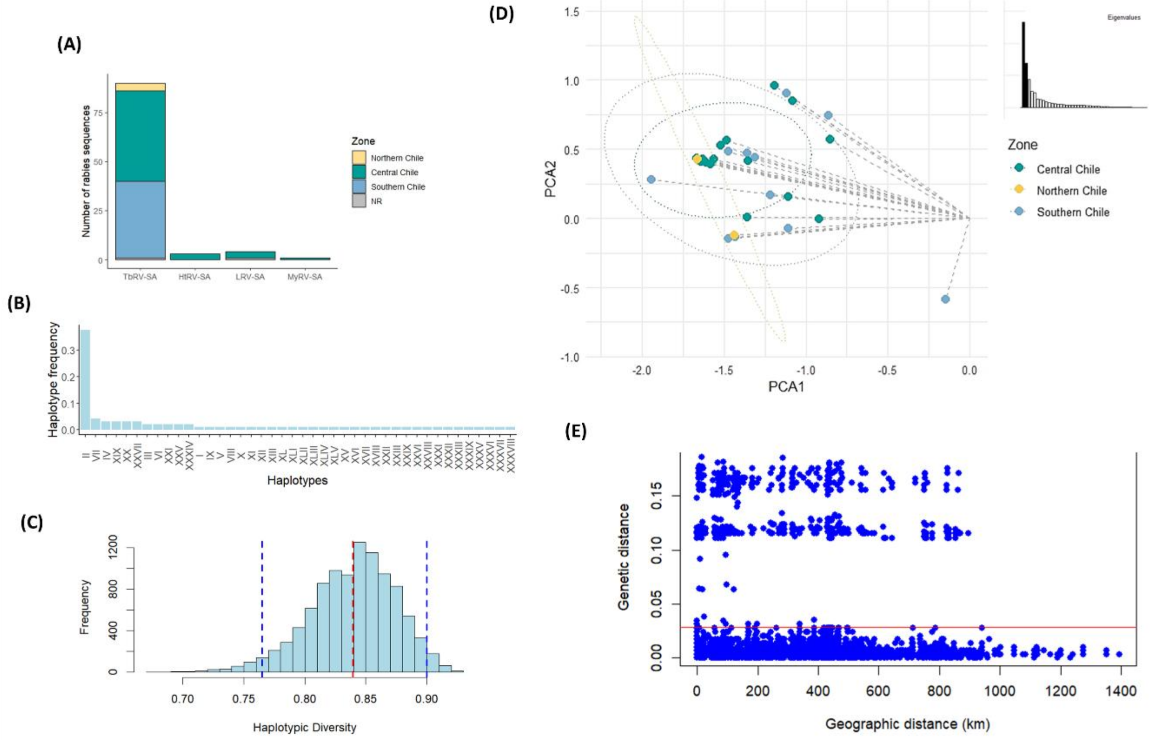

S3 Fig. Regional distribution and genetic analyses of *Tadarida brasiliensis* of rabies lineages in Chile. (**A**) Bars represent the total number of rabies virus sequences per bat-rabies lineage by zone. Each bar represents a bat-rabies lineage: *Tadarida* Rabies Virus-South America (TbRV-SA), *Lasiurus* Rabies Virus-South America (LRV-SA), *Histiotus* Rabies Virus-South America (HtRV-SA), *Myotis* Rabies Virus-South America (MyRV-SA). Chilean zones were divided into northern (yellow), central (green), and southern (cyan-blue). Samples without geographic distribution were classified as non-reported (NR, grey). (**B**). Bars represent haplotype frequency. (**C**). Bars represent the bootstrap distribution of haplotypic diversity. Dashed lines in blue represent the 95% confidence intervals. The dashed red line represents the mean bootstrap value (bv = 0.84, IC 95%= 0.77-0.90). (**D**). Discriminant analysis of principal components for *T. brasiliensis* rabies virus sequences. The main plot demonstrates the first two discriminant axes (ANOSIM test, R = 0.0008, p-value

= 0.370). Color represents Chilean Zones. The secondary plot inserted in the top left displays the percentage of variation by component. (**E**). Correlation between pairwise genetic distances and pairwise geographic distances for *T. brasiliensis* rabies virus. The red line represents the tendency of the correlation.
