# Supplementary material for: Rabies, host population structure, and cross-species transmission to the migratory bat Tadarida brasiliensis in Chile
Source: PLoS Negl Trop Dis. 2026 Feb 19;20(2):e0013964. doi: 10.1371/journal.pntd.0013964 (PMC12919816; doi:10.1371/journal.pntd.0013964)
Supplement: S4 Fig — Links connect each TbRV-SA sequence to a T. brasiliensis cytochrome b sequence from the same Chilean zone. Link colors indicate the Chilean zone of origin. Chilean zones were divided into northern (yellow), central (green), and southern (cyan-blue). (PDF) [file pntd.0013964.s004.pdf]

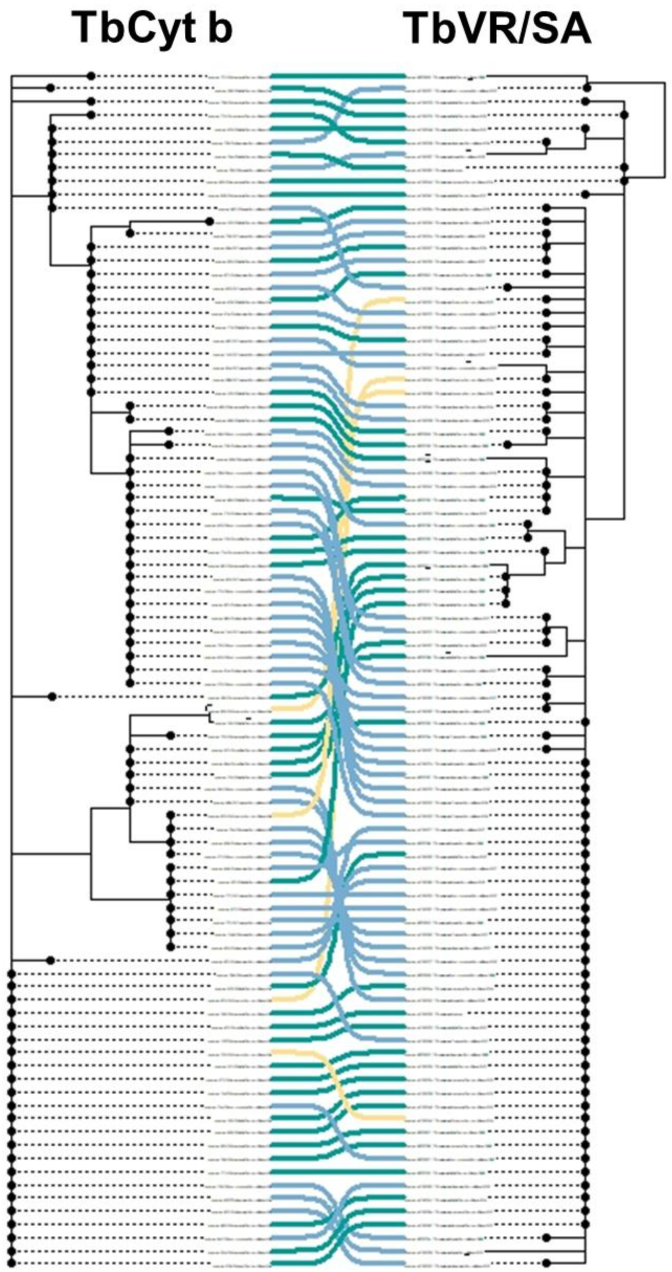

S4 Fig. Tanglegram shows the relationship between *T. brasiliensis* cytochrome b (TbCytB) and *Tadarida* Rabies Virus-South America (TbRV-SA) sequences. Links connect each TbRV-SA sequence to a *T. brasiliensis* cytochrome b sequence from the same Chilean zone. Link colors indicate the Chilean zone of origin. Chilean zones were divided into northern (yellow), central (green), and southern (cyan-blue).
