# Supplementary material for: Rabies, host population structure, and cross-species transmission to the migratory bat Tadarida brasiliensis in Chile
Source: PLoS Negl Trop Dis. 2026 Feb 19;20(2):e0013964. doi: 10.1371/journal.pntd.0013964 (PMC12919816; doi:10.1371/journal.pntd.0013964)
Supplement: S5 Fig — (A). Lasiurus Rabies Virus-South America (LRV-SA) lineage sample distribution. (B). Myotis Rabies Virus-South America (MyRV-SA) lineage sample distribution. (C). Histiotus Rabies Virus-South America (HtRV-SA) lineage sample distribution. The dot color and shape represent the bat species sampled and zone distribution. Chilean zones were divided into northern (yellow), central (green), and southern (cyan-blue). Base layer map was obtained from the open-source site Global Administrative Areas (GADM) website (https://gadm.org/) using geodata package in R [71]. (PDF) [file pntd.0013964.s005.pdf]

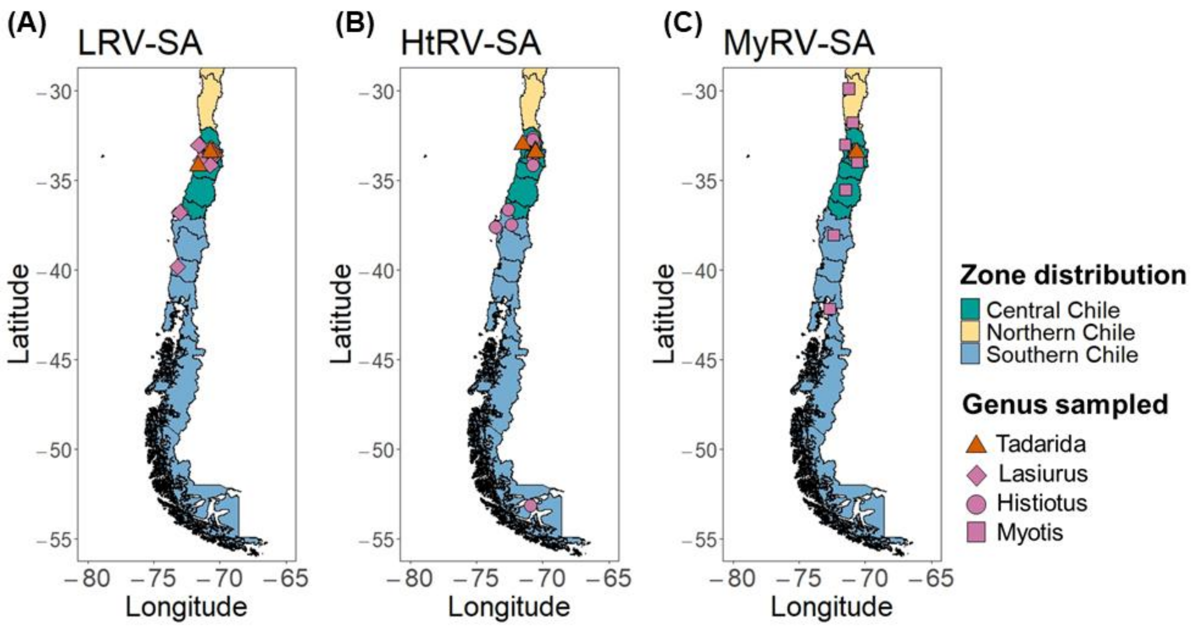

S5 Fig. Location of non-*Tadarida* rabies virus in Chile. **(A)**. *Lasiurus* Rabies Virus-South America (LRV-SA) lineage sample distribution. **(B)**. *Myotis* Rabies Virus-South America (MyRV-SA) lineage sample distribution. **(C)**. *Histiotus* Rabies Virus-South America (HtRV-SA) lineage sample distribution. The dot color and shape represent the bat species sampled and zone distribution. Chilean zones were divided into northern (yellow), central (green), and southern (cyan-blue). Base layer map was obtained from the open-source site Global Administrative Areas (GADM) website (https://gadm.org/) using geodata package in R [71].
