# Supplementary material for: Rabies, host population structure, and cross-species transmission to the migratory bat Tadarida brasiliensis in Chile
Source: PLoS Negl Trop Dis. 2026 Feb 19;20(2):e0013964. doi: 10.1371/journal.pntd.0013964 (PMC12919816; doi:10.1371/journal.pntd.0013964)
Supplement: S1 Table — H: haplotype diversity, CI: Confidence interval, N: nucleotide diversity, SD: standard deviation, TbRV-SA: Tadarida Rabies Virus-South America. (PDF) [file pntd.0013964.s006.pdf]

S1 Table. Distribution of *Tadarida* and non-*Tadarida* rabies virus in Chile.

**Sequences**	**Chilean Zones**	**Number of Haplotypes**	**Haplotype diversity (H (95% CI))**	**Nucleotide diversity (N±SD)**
**Cytochrome b**	All Zones	28	0.9 (0.88–0.91)	0.005± 0.001
	Central Chile	18	0.9 (0.82-0.90)	0.005± 0.002
	Southern Chile	15	0.8 (0.70-0.88)	0.004± 0.001
	Northern Chile	9	0.8 (0.70-0.86)	0.005± 0.002
**TBRV-SA**	All Zones	45	0.9 (0.77–0.90)	0.02 ± 0.002
	Central Chile	22	0.8 (0.67-0.88)	0.005 ± 0.001
	Southern Chile	19	0.8 (0.66-0.88)	0.007±0.002
	Northern Chile	4	0.7 (0.38-0.80)	0.02±0.005

H: haplotype diversity, CI: Confidence interval, N: nucleotide diversity, SD: standard deviation, TbRV-SA: *Tadarida* Rabies Virus-South America.
