# Supplementary material for: Rabies, host population structure, and cross-species transmission to the migratory bat Tadarida brasiliensis in Chile
Source: PLoS Negl Trop Dis. 2026 Feb 19;20(2):e0013964. doi: 10.1371/journal.pntd.0013964 (PMC12919816; doi:10.1371/journal.pntd.0013964)
Supplement: S2 Table — HtRV-SA: Histiotus Rabies Virus-South America, LRV-SA: Lasiurus Rabies Virus-South America, MyRV-SA: Myotis Rabies Virus-South America, TbRV-SA: T. brasiliensis Rabies Virus-South America. Samples without geographic distribution were classified as non-reported (NR). (PDF) [file pntd.0013964.s007.pdf]

S2 Table. Distribution of *Tadarida* and non-*Tadarida* rabies virus in Chile.

			**Chilean Zones**				
**Lineage**	**Genus**	**Species**	**Central Chile**	**Northern Chile**	**Southern Chile**	**NR**	**Total**
HtRV-SA	*Histiotus*	*H. macrotus*	9				9
		Histiotus sp.	2		6	1	9
		Histiotus Total	11		6	1	18
	Tadarida	*T. brasiliensis*	3				3
	HtRV-SA Total		14		6	1	21
LRV-SA	*Lasiurus*	*L. borealis*	2		2		4
		L. cinereus	9		1		10
		Lasiurus sp.	4			1	5
		Lasiurus Total	15		3	1	19
	Tadarida	*T. brasiliensis*	3			1	4
	LRV-SA Total		18		3	2	23
MyRV-SA	*Myotis*	*M. chiloensis*	3	2	2		7
		Myotis sp.	1			1	2
		Myotis Total	4	2	2	1	9
	Tadarida	*T. brasiliensis*	1				1
	MyRV-SA Total		5	2	2	1	10
TbRV-SA	*Tadarida*	*T. brasiliensis*	46	4	39	1	90
	TbRV-SA Total		46	4	39	1	90
		**Total**	83	6	50	5	144

HtRV-SA: *Histiotus* Rabies Virus-South America, LRV-SA: *Lasiurus* Rabies Virus-South America, MyRV-SA: *Myotis* Rabies Virus-South America, TbRV-SA*: T. brasiliensis* Rabies Virus-South America. Samples without geographic distribution were classified as non-reported (NR).
